# Impact of Joint Line Displacement on Function and Quality of Life After Primary Total Knee Arthroplasty

**DOI:** 10.3390/jcm15103737

**Published:** 2026-05-13

**Authors:** Eugenio Miguel Ferrer Santacreu, Sara López Resino, Yentl Garcelán Pecharromán, Pablo Cendrero Cendrero

**Affiliations:** 1 Orthopaedic Surgery and Traumatology Service, Hospital Universitario de Móstoles, 28935 Móstoles, Madrid, Spain; yentlgp@gmail.com (Y.G.P.); pcendrero2@gmail.com (P.C.C.); 2 Faculty of Health Sciences, Universidad Rey Juan Carlos, 28922 Alcorcón, Madrid, Spain; 3 Geriatrics Service, Hospital Universitario de Getafe, 28905 Getafe, Madrid, Spain; sara.lopezr5@gmail.com

**Keywords:** total knee arthroplasty, joint line, functional outcomes, quality of life, knee biomechanics, radiographic assessment

## Abstract

**Background/Objectives:** Total knee arthroplasty (TKA) is one of the most common surgeries among people over 60. Joint line restoration plays an important role in knee biomechanics, with joint line elevation or depression after TKA being associated with poorer postoperative outcomes, although there is no consensus regarding the threshold at which these variations become clinically relevant. The objectives of this study were to evaluate whether a joint line variation greater than 4 mm after primary TKA affects postoperative outcomes, and to assess the concordance between different radiographic methods used to measure joint line height. **Methods:** A retrospective study was conducted including patients over 60 who underwent primary TKA for knee osteoarthritis. Joint line height variations were evaluated preoperatively and postoperatively using three radiographic measurements: lateral femoral epicondyle–fibular head (LEFH) distance, adductor tubercle–joint line (ATJL) distance, and Blackburne–Peel index. Quality of life was assessed using the Short Form-12 (SF-12) questionnaire, and functionality using the Knee Society Score (KSS). Statistical analysis was carried out using R software. **Results:** Seventy-three patients were included. No statistically significant associations were found between joint line displacement and functional outcomes (KSS), quality of life (SF-12), or postoperative complications. Concordance analysis between radiographic methods showed a significant but weak correlation between the LEFH and ATJL measurements (ρ = 0.419; *p* < 0.001). **Conclusions:** Joint line displacement after primary TKA was not associated with poorer postoperative outcomes in this cohort. These findings suggest that its clinical impact may depend more on its magnitude than on its mere presence and may also be influenced by additional factors. The weak concordance observed between radiographic measurement methods highlights the need for standardized criteria.

## 1. Introduction

Osteoarthritis of the knee is one of the world’s most prevalent conditions and one of the leading causes of chronic pain, impaired mobility, and diminished quality of life in the elderly [1]. As a degenerative disease, it constitutes the most common indication for total knee arthroplasty (TKA), with 97.4% of cases being attributable to the condition [2,3]. The progressive aging of the population, together with a growing number of seniors demanding more active lifestyles, has notably increased the number of TKAs performed in the last few decades [4]. The procedure is one of the most frequent among people above the age of 60, with increasingly favorable clinical and functional results thanks to the development of increasingly sophisticated implants and surgical techniques [5,6].

Nevertheless, in spite of these advances, it has been estimated that between 20% and 30% of operated patients fail to achieve the expected degree of satisfaction following TKA [4]. This may be explained by various factors, both patient-related (expectations, age, comorbidities), and procedure-related. The latter factors include the precision of bone cuts; implant positioning and orientation; lower limb alignment; and soft tissue balancing, both in flexion and in extension. In addition, joint line restoration, which has received widespread attention in the literature, is another mainstay for proper reconstruction of knee biomechanics [7,8].

It has indeed been demonstrated that the joint line plays a key role both in tibiofemoral motion and in patellofemoral joint biomechanics [9]. A proximally displaced joint line (joint line elevation) could result in a secondary patella baja, also known as a pseudo-patella baja, which typically induces impingement and anterior pain. A distally displaced joint line (joint line depression), on the other hand, is associated with patella alta, disturbances in the extensor mechanism, and a risk of patellar subluxation [10]. It has been reported that variations equal to or higher than 4 mm in the height of the joint line may result in midflexion instability and poorer overall knee function [7,11,12,13].

It must be said that a marked displacement of the joint line is not always associated with poorer clinical outcomes: the observed displacement may in some cases result from correction of an abnormal preoperative joint line, which should contribute to improved joint function. This observation adds complexity to the interpretation of this parameter and has contributed to a lack of consensus over what degree of displacement should be considered clinically significant [7]. Moreover, the existence of several radiological methods for measuring the position of the joint line makes it more difficult to establish comparisons across studies and to extrapolate their clinical results, as there is no universally accepted standard [11,14].

Against this background, it is of particular interest to analyze the impact of joint line displacement on patients’ clinical evolution following primary TKA. We hypothesized that a displaced joint line after primary TKA would be associated with worse functional outcomes and quality of life. The present study aimed to contribute some evidence on a matter fraught with controversy and over which no standard has yet been established. An evaluation was made of the relationship between joint line displacement and clinical/functional outcomes as well as the concordance between the different radiological methods employed for measuring joint line height.

## 2. Materials and Methods

This retrospective observational study included all consecutive patients diagnosed with osteoarthritis of the knee who underwent a TKA at Mostoles University Hospital (Spain) between September 2019 and June 2023 and met the selection criteria. The study protocol was approved by the Hospital’s Institutional Review Board (approval code 37/2023). All patients were required to give their informed consent prior to being enrolled.

To be included in the study, patients had to be aged over 60 years and be diagnosed with osteoarthritis of the knee. All patients had to have been operated on by the same surgeon, with the same prosthetic system (Legion, Smith & Nephew PLC, Watford, UK), and had to have been followed up for at least one year. Patients who underwent bilateral procedures were excluded.

A record was made of sociodemographic variables (age, sex, body mass index (BMI) and smoking status), as well as of variables related to the patients’ condition (operated knee), the surgical procedure (patellar resurfacing), postoperative outcomes (complications; function as measured by the Knee Society Score (KSS) through its KSS knee and KSS function subscales, and quality of life, as measured by the Short Form-12 (SF-12) questionnaire’s physical and mental components), and radiographic variables (joint line height variation).

**Figure 1:**
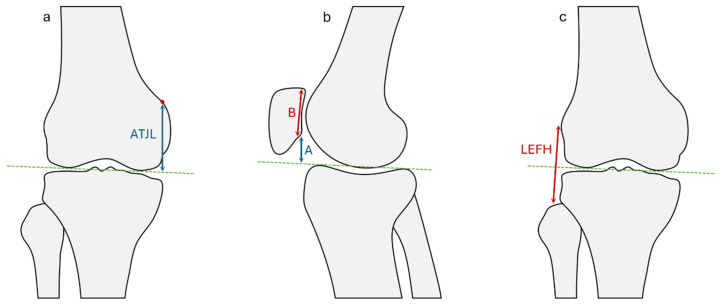
Methods employed for measuring variations in joint line height: (**a**) adductor tubercle-joint line (ATJL) distance; (**b**) Blackburne–Peel index: relationship between the height of the inferior border of the patellar articular surface (A) and the articular surface length of the patella (B); (**c**) lateral femoral epicondyle–fibular head (LEFH) distance. The green dashed line represents the knee joint line.

Joint line height variations were evaluated using three radiographic measurements, performed before and after surgery (Figure 1):

1. Lateral femoral epicondyle–fibular head (LEFH) distance: these landmarks, commonly used in the literature for joint line assessment [15,16], were used together in this study as an indirect measurement of joint line variation. Any variation in excess of 4 mm with respect to the preoperative value was considered a significant abnormality, the joint line being classified as elevated when the postoperative LEFH distance was more than 4 mm shorter than preoperatively; depressed when the postoperative distance was more than 4 mm longer; or maintained when the difference was between −4 and +4 mm.
2. Adductor tubercle–joint line (ATJL) distance [17,18]: the same criteria were applied as when measuring the LEFH distance.
3. Blackburne–Peel index [19]: values below 0.5 (patella baja) and above 1 (patella alta) were considered abnormal. Values between 0.5 and 1 were considered normal.

The statistical analysis was carried out using R software (R Development Core Team, Vienna, Austria, version 4.4.3) [20]. Central tendency (mean, median) and dispersion (standard deviation, range) were calculated for quantitative variables, and absolute and relative frequencies were used for qualitative variables. Associations between qualitative variables were evaluated using the Pearson chi-squared test or Fisher’s Exact Test, depending on the magnitude of the expected values. Correlations between continuous variables were analyzed by means of Spearman’s correlation coefficient. The differences between the quantitative variables of the two groups were evaluated using Student’s *t* test or the Wilcoxon test, depending on the distribution of data. Comparisons between the variables of three or more groups were carried out using the analysis of variance (ANOVA) test or the Kruskal–Wallis test, as appropriate. A concordance analysis was also performed between the different radiographic methods employed to measure the joint line. Statistical significance was set at a *p* value < 0.05.

### Surgical Technique

The same surgical technique was used in all cases. The preoperative plan was carried out using TraumaCad software (BrainLab AG, Munich, Germany), which made it possible to estimate the level at which the femoral and distal osteotomies had to be carried out and to determine the size of the prosthetic components required. During surgery, a mechanical alignment technique was used [21,22], which involved a distal femoral osteotomy with an intramedullary guide, typically positioned at 6° valgus unless otherwise provided by the preoperative plan. The femoral preparation was completed with posterior referencing and a cutting block adapted to the size of the prosthetic components employed, maintaining 3° of external rotation with respect to the posterior condylar plane. An intramedullary guide was used to perform a 3–6 mm osteotomy at the portion of the tibial plateau exhibiting the greatest amount of wear, in accordance with the preoperative plan, subsequently creating a 3-degree posterior tibial slope.

## 3. Results

A total of 73 patients were included in the study, 49 females (67.1%) and 24 males (32.9%), with a mean age of 73.63 ± 7.20 years (range: 60–89). Mean bone mass index (BMI) was 29.92 ± 5.15 kg/m^2^ (range: 20.5 a 42.8), and 12.3% of patients were smokers. A total of 38 TKAs were performed on the left knee and 35 on the right knee. Only 10 cases (13.7%) included resurfacing of the patella. During follow-up, 23 patients (29.1%) reported mechanical pain during ambulation or when climbing or going downstairs (complications) (Table 1).

As regards postoperative joint line displacement, 43 of the 73 patients (58.9%) presented with a postoperative displacement in excess of 4 mm, as measured by the LEFH distance. In 13 of these cases (17.8%), the joint line was elevated, and in 30 (41.1%), it was depressed. According to the ATJL distance measurements, 31 patients (42.5%) experienced a displacement of more than 4 mm, 17 of whom presented with an elevated joint line and 14 with a depressed one. As regards the Blackburne–Peel index, 19 patients (26.03%) presented with abnormal postoperative values, with 4 cases of patella alta and 15 of patella baja. In sum, a total of 8 patients (11%) presented with abnormal values in all three of the parameters considered (Table 2).

As far as the functional and quality-of-life scales are concerned, the mean score on the KSS knee scale of the Knee Society Score (KSS) was 71.68 ± 18.31 points, while the mean score on the KSS function scale was 68.42 ± 22.51 points. The SF-12 questionnaire’s physical component yielded a mean of 38.00 ± 11.30 points, whereas the mean score on the mental component was 47.00 ± 13.51 points.

**Figure 2:**
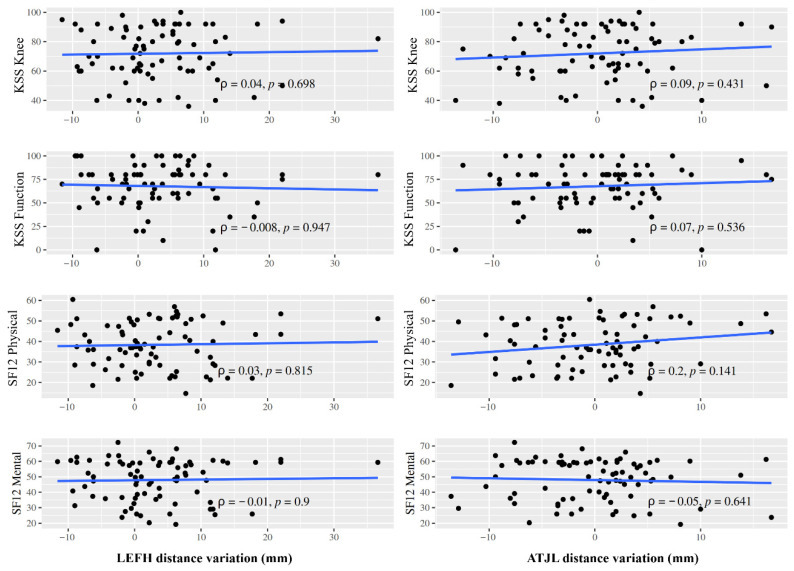
Spearman’s correlations between pre- and postoperative joint line height values, as measured using the LEFH distance and the ATJL distance, and Knee Society Score (KSS) and Short Form-12 (SF-12) scores.

An analysis of the association between those scales and the variations in joint line height taken as a continuous value using Spearman’s correlation test did not reveal any statistically significant correlation between the degree of displacement and the postoperative KSS and SF-12 scores on any of the parameters employed (*p* > 0.05) (Figure 2).

Similarly, no statistically significant difference was observed with respect to function and quality of life scores. Such scores were similar across the groups, regardless of postoperative joint line (elevated/maintained/depressed) or patellar position (high-riding, normal, low-riding) (*p* > 0.05). The means of the different categories, represented in Table 3, exhibited very similar values, without any trend or variation suggesting a relationship between the postoperative position of the joint line and clinical/functional outcomes.

An analysis of joint line displacement and the appearance of postoperative complications did not reveal any significant associations either, regardless of the measurement method employed (*p* > 0.05). When using the LEFH distance, complications occurred in 30.8% of patients with an elevated joint line, in 30.0% of those with a maintained joint line, and in 33.3% of those with a depressed joint line (*p* = 1). Similar results were obtained when using the ATJL distance (*p* = 0.229) and the Blackburne–Peel index (*p* = 0.057) (Table 3).

In addition, an evaluation was made of the influence of other variables recorded in the study on the patients’ KSS and SF-12 scores. Statistically significant differences were observed between males and females, such that males exhibited significantly higher scores on the KSS function scale and the SF-12 questionnaire’s physical and mental components. Moreover, age was significantly correlated with the KSS function scale, with scores being consistently lower in older patients. Other variables such as BMI, smoking status, undergoing patellar resurfacing or experiencing complications did not show a significant correlation with KSS or SF-12 scores. Results are shown in Table 4.

Lastly, the concordance between the different radiographic techniques for measuring joint line displacement was determined by analyzing the postoperative joint line displacements obtained by measuring the LEFH distance and the ATJL distance. A weak yet significant positive correlation was observed between both measurements (ρ = 0.419; *p* < 0.001). This weak correlation was reflected in a means difference analysis, which yielded significantly disparate values (mean: −3.4 ± 8.06 mm; *p* < 0.001).

## 4. Discussion

The present study analyzed the relationship between joint line displacement following primary TKA and the patients’ long-term clinical/functional outcomes, as evaluated by the Knee Society Score (KSS) and the SF-12 questionnaire. The analysis distinguished between postoperative joint line elevation, maintenance or depression, using three different radiographic methods: the LEFH distance, the ATJL distance and the Blackburne–Peel index, the latter serving as a surrogate indicator for the position of the patella. No statistically significant differences were found on the KSS or SF-12 scores between patients with an elevated, a maintained or a depressed postoperative joint line, nor were any significant correlations observed between the degree of displacement and the patients’ clinical outcomes. These findings suggest that joint line alterations of the magnitudes observed in the study do not seem to, at least significantly, impact the patients’ long-term function or quality of life.

The findings of this study are in line with a significant portion of the available evidence. Yang et al. [23] found no significant differences in clinical outcomes between patients with a postoperative joint line displacement higher or lower than 3 mm. Similarly, Clavé et al. [11], only found a weak correlation between joint line elevation (>4 mm) and the KSS function score and no correlation whatsoever with the KSS knee score. Other authors were also unable to find an association between the position of the joint line and function, pain or quality of life following TKA [9,10,24,25]. Even in studies comparing various surgical techniques—such as “gap balancing” vs. “measured resection”—failure to restore the patients’ preoperative joint line height did not result in differences in the patients’ function or quality of life [26]. In summary, it can be said that most authors in the literature fail to establish a conclusive relationship between joint line displacement and clinical/functional outcomes following TKA, particularly in studies where joint line alteration is defined using relatively low thresholds (approximately 3–5 mm).

However, no universally agreed-on consensus exists on the subject. Several authors have described a negative relationship between excessive joint line elevation and functional outcomes, particularly when the elevation is above 4–5 mm, a threshold considered clinically relevant by most literature reviews [27,28,29]. Van Lieshout et al. [7] concluded that an elevation over 4 mm is associated with poorer KSSs and recommended avoiding larger displacements during surgery. Similarly, Koshire et al. [4] found elevations greater than or equal to 5 mm to be correlated with poorer functional outcomes and more restricted mobility, as reflected by lower KSS and Western Ontario and McMaster Universities Osteoarthritis Index (WOMAC) scores. In the same vein, Partington et al. [30] reported that significant joint line elevations, in particular those in excess of 8 mm, are correlated with significantly lower clinical outcomes following revision arthroplasty. The literature suggests that the clinical impact of joint line displacement does not depend so much on the fact that a displacement has occurred, but on the magnitude of such a displacement. Nevertheless, there is still no consensus regarding the exact displacement beyond which clinically significant function or quality of life differences start appearing [7]. In the present study, no statistically significant association was observed between joint line alteration and the patients’ KSS or the SF-12 scores using a threshold of 4 mm.

Despite the clinical importance traditionally attributed to proper joint line restoration, one of the main reasons behind the disparity across the different studies is the absence of a standardized method to measure joint line displacement [31,32]. Multiple techniques have been developed to quantify joint line displacements following TKA, although no universally accepted gold standard [11,14]. Sadaka et al. [33] pointed out that, although several anatomical and radiographic methods have been proposed, none of them has been shown to be consistently reliable. The most commonly used anatomical landmarks include the medial epicondyle, the fibular head, the inferior pole of the patella and particularly the adductor tubercle, regarded by many authors as the most precise, reproducible and easily identifiable landmark both in radiographs and during surgery [17,34]. These authors showed the ATJL distance to be well correlated with the subjects’ native joint line height and recommended it be used to facilitate restoration of the joint line in primary and revision surgery. Moreover, the adductor tubercle tends to remain intact following surgery, as opposed to other anatomical landmarks, which may undergo changes or even disappear [33].

More advanced methods have recently been put forward for improving the reliability and reproducibility of joint line height measurements. Some authors have proposed methods involving ratios between bone structures, such as the Adductor Tubercle Ratios Method [35], or other formulas based on the width of the femur [36], to reduce the potential errors resulting from radiographic magnification and inter-individual anatomical variability. Other studies have proposed three-dimensional techniques based on surface scanning or 3D registration methods [37,38]. However, despite these advances there is as yet no consensus in the literature as to the most precise or clinically relevant method for evaluating joint line displacement [32,39]. In view of the lack of an agreed gold standard, measurements in the present study were made using three different radiographic methods, with a view to analyzing their concordance and determining which of them provided a more reliable estimation in clinical practice. Among the available methods, the Blackburne–Peel index was selected due to its routine use in our clinical practice, while the ATJL distance was selected based on its established reliability and reproducibility in the literature [17,34]. In addition, the LEFH distance was incorporated as an indirect measurement of joint line variation based on anatomical landmarks widely used in the literature [15,16].

Consequently, in addition to quantifying the joint line displacements experienced by our subjects, the study set about evaluating the concordance between two radiographic methods: the LEFH distance and the ATJL distance. The analysis revealed a weak yet statistically significant positive correlation (ρ = 0.419; *p* < 0.001), which means that, although both methods were found to measure some common features of joint line displacement, the correlation between them is not strong enough to consider them interchangeable. In this respect, the means difference analysis performed as part of this study identified statistically significant discrepancies between the measurements made (–3.4 ± 8.06 mm; *p* < 0.001). Although previous studies have analyzed the reliability of various anatomical landmarks for measuring the joint line [32,40,41,42], none of them has to the best of our knowledge made a head-to-head comparison of these two techniques in one single cohort. Our results suggest that both methods offer partially overlapping estimates, although the magnitude of the difference observed could lead to an over- or underestimation of joint line displacement if one single method was applied in isolation. In this regard, anatomical landmark selection should be conducted with caution and, where possible, be supplemented by other measurement techniques.

Although no significant association was found in our series between the degree of joint line displacement and functional outcomes, quality of life or complications, this finding should be interpreted in its appropriate context. Indeed, the clinical impact in most cases considered—where joint line displacements stood within a moderate range—may be of limited extent and be influenced by other factors. Overall limb alignment, prosthetic design, ligament balancing and the patients’ characteristics (age, sex, preoperative function) have been described in the literature as potential determinants of post-TKA outcomes [8,43,44,45,46]. Our study also identified associations between demographic variables and functional and quality of life scores. Specifically, statistically significant differences were observed between males and females, both on the KSS function scale and the SF-12 questionnaire’s physical and mental components, women exhibiting lower scores. This pattern was also observed by other authors [44,47]. Furthermore, age showed itself to be significantly associated with KSS function scores, with older patients showing poorer outcomes. In this respect, the literature presents contrasting findings [48,49]. Further research should focus not only on the displacement of the joint line but also on the factors that could modulate the effect of any changes in joint line height.

The present study is not exempt from limitations. First and foremost, its retrospective design, which precluded an a priori sample size calculation, and the absence of preoperative KSS and SF-12 scores preclude an individualized assessment of the evolution of patient function. Secondly, although three radiographic methods were analyzed, only concordances between two of them were analyzed, as the Blackburne–Peel index does not allow direct expression of displacement in millimeters. In addition, technical factors such as radiographic magnification, the position of the knee while images are acquired and the lack of a universal gold standard could have influenced the measurements obtained. Finally, the small size of our sample (*n* = 73) and the choice of an arbitrary cut-off point (>4 mm) make it difficult to generalize our results.

## 5. Conclusions

The present study did not find a direct relationship between joint line displacement greater than 4 mm and the clinical/functional outcomes obtained following TKA. These findings highlight the importance of considering the magnitude of joint line displacement, rather than its mere presence, when evaluating postoperative outcomes, while also taking into account other patient- and procedure-related factors that may influence clinical results. Even so, the lack of a universally accepted measuring method, compounded by a significant amount of inter-observer variability, remains a significant challenge. Consequently, it is paramount to develop standardized radiographic criteria that allow more accurate inter-study comparisons giving rise to conclusions that can be successfully applied in clinical practice.

## Figures and Tables

**Table 1:** Descriptive variables of the sample. Demographic, clinical and functional characteristics.

**Variable**	***N***	**Mean ± SD**	**Median**	**Minimum**	**Maximum**
Age	73	73.63 ± 7.20	75	60	89
BMI	73	29.92 ± 5.15	29.10	20.50	42.80
**Variable**	**Category**		**Frequency**		**%**
Sex	Female		49		67.1
	Male		24		32.9
Smoker	No		64		87.7
	Yes		9		12.3
Knee	Right		35		47.9
	Left		38		52.1
Patellar resurfacing	No		63		86.3
	Yes		10		13.7
Complications	No		50		68.5
	Yes		23		31.5

**Table 2:** Postoperative status of the tibiofemoral joint line according to the three radiographic methods employed.

Parameter	Result	Frequency	%
Lateral femoral epicondyle–fibular head distance	Elevated joint line	13	17.81
	Maintained joint line	30	41.10
	Depressed joint line	30	41.10
Adductor tubercle–joint line distance	Elevated joint line	17	23.29
	Maintained joint line	42	57.53
	Depressed joint line	14	19.18
Blackburne–Peel index	Patella alta	4	5.48
	Normal	54	73.97
	Patella baja	15	20.55
Abnormal values across all parameters	No	65	89
	Yes	8	11

**Table 3:** KSS and SF-12 scores and distribution of complications according to the postoperative position of the joint line according to the different measurement methods employed.

	**Lateral Femoral Epicondyle–Fibular Head Distance**			
**Scales**	**Elevated joint line (<−4)**	**Maintained joint line (−4 a 4)**	**Depressed joint line (>4)**	***p* value**
KSS knee	71.31 ±18.62	70.37 ± 18.19	73.17 ± 18.82	0.84
KSS function	69.62 ± 27.80	66.33 ± 19.12	70.00 ± 23.78	0.806
SF-12 physical	39.03 ± 11.69	36.85 ± 8.87	38.70 ± 13.40	0.771
SF-12 mental	49.18 ± 10.67	44.79 ± 14.21	48.26 ± 13.99	0.502
Complications	4 YES/9 NO	9 YES/21 NO	10 YES/20 NO	1
	**Adductor tubercle–joint line distance**			
**Scales**	**Elevated joint line (<−4)**	**Maintained joint line (−4 a 4)**	**Depressed joint line (>4)**	***p* value**
KSS knee	69.82 ± 17.45	73.64 ± 18.24	68.07 ± 20.07	0.555
KSS function	65.59 ± 26.27	69.64 ± 19.80	68.21 ± 26.57	0.825
SF-12 physical	36.16 ± 11.10	37.55 ± 10.75	41.59 ± 13.13	0.386
SF-12 mental	48.73 ± 14.70	46.97 ± 12.94	44.97 ± 14.45	0.661
Complications	4 YES/13 NO	12 YES/30 NO	7 YES/7 NO	0.229
	**Blackburne–Peel index**			
**Scales**	**Patella baja (<0.5)**	**Normal patella** **(0.5 a 1)**	**Patella alta (>1)**	***p* value**
KSS knee	76.33 ± 16.61	69.89 ± 18.33	78.50 ± 24.41	0.291
KSS function	67.33 ± 26.31	68.24 ± 21.26	75.00 ± 29.72	0.831
SF-12 physical	40.07 ± 11.11	37.17 ± 11.32	41.36 ± 13.31	0.57
SF-12 mental	48.58 ± 13.41	46.39 ± 13.58	49.19 ± 16.16	0.814
Complications	8 YES/7 NO	13 YES/41 NO	2 YES/2 NO	0.057

**Table 4:** Associations between sociodemographic, clinical and surgical variables and KSS and SF-12 scores.

Variables	KSS Knee (*p*)	KSS Function (*p*)	SF-12 Physical (*p*)	SF-12 Mental (*p*)
Age	0.564	0.018 *	0.827	0.305
BMI	0.267	0.297	0.205	0.131
Sex	0.414	0.01 *	0.006 *	0.046 *
Smoking status	0.790	0.824	0.875	0.690
Patellar resurfacing	0.209	0.831	0.750	0.548
Complications	0.812	0.289	0.411	0.929

Note: * Significant values (*p* < 0.05).

## Data Availability

The data presented in this study are available on request from the corresponding author due to privacy or ethical restrictions.

## Abbreviations

The following abbreviations are used in this manuscript:

ANOVA	Analysis of variance
ATJL	Adductor tubercle–joint line
BMI	Body mass index
KSS	Knee Society Score
LEFH	Lateral femoral epicondyle–fibular head
SF-12	Short Form-12
TKA	Total knee arthroplasty
WOMAC	Western Ontario and McMaster Universities Osteoarthritis Index
